# Yield of the electrophysiological study in patients with new-onset left bundle branch block after transcathether aortic valve replacement: The PR interval matters

**DOI:** 10.3389/fcvm.2022.910693

**Published:** 2022-09-06

**Authors:** Mattia Pagnoni, David Meier, Adrian Luca, Stephane Fournier, Farhang Aminfar, Pascale Gentil, Christelle Haddad, Giulia Domenichini, Mathieu Le Bloa, Claudia Herrera-Siklody, Stephane Cook, Jean-Jacques Goy, Christan Roguelov, Grégoire Girod, Vladimir Rubimbura, Marion Dupré, Eric Eeckhout, Etienne Pruvot, Olivier Muller, Patrizio Pascale

**Affiliations:** ^1^Department of Cardiology, Lausanne University Hospital, Lausanne, Switzerland; ^2^Division of Cardiology, Department of Advanced Biomedical Sciences, University of Naples Federico II, Naples, Italy; ^3^Arrhythmias Unit, Louis Pradel Cardiovascular Hospital, Hospices Civils de Lyon, Lyon, France; ^4^Department of Cardiology, Clinique Cecil Hirslanden Group, Lausanne, Switzerland; ^5^Department of Cardiology, University Hospital Fribourg, Fribourg, Switzerland

**Keywords:** electrophysiological study (EPS), trans-catheter aortic valve replacement (TAVR), atrioventricular block (AV block), HV interval, PR interval

## Abstract

**Background:**

Studies suggest that performing an electrophysiological study (EPS) may be useful to identify patients with new-onset left bundle branch block (LBBB) post-TAVR at risk of atrioventricular block. However, tools to optimize the yield of such strategy are needed. We therefore aimed to investigate whether 12-lead ECG changes post-TAVR may help identify patients with abnormal EPS findings.

**Materials and methods:**

Consecutive patients with new-onset LBBB post-TAVR who underwent EPS were included. PR and QRS intervals were measured on 12-lead ECG pre-TAVR and during EPS. Abnormal EPS was defined as an HV interval > 55 ms.

**Results:**

Among 61 patients, 28 (46%) had an HV interval > 55 ms after TAVR. Post-TAVR PR interval and ΔPR (PR-post–pre-TAVR) were significantly longer in patients with prolonged HV (PR: 188 ± 38 vs. 228 ± 34 ms, *p* < 0.001, ΔPR: 10 ± 30 vs. 34 ± 23 ms, *p* = 0.001), while no difference was found in QRS duration. PR and ΔPR intervals both effectively discriminated patients with HV > 55 ms (AUC = 0.804 and 0.769, respectively; *p* < 0.001). A PR > 200 ms identified patients with abnormal EPS results with a sensitivity of 89% and a negative predictive value (NPV) of 88%. ΔPR ≥ 20 ms alone provided a somewhat lower sensitivity (64%) but combining both criteria (i.e., PR > 200 ms *or* ΔPR ≥ 20 ms) identified almost every patients with abnormal HV (sensitivity = 96%, NPV = 95%). Selecting EPS candidate based on both criteria would avoid 1/3 of exams.

**Conclusion:**

PR interval assessment may be useful to select patients with new-onset LBBB after TAVR who may benefit most from an EPS. In patients with PR ≤ 200 ms *and* ΔPR < 20 ms the likelihood of abnormal EPS is very low independently of QRS changes.

## Introduction

Trans-catheter aortic valve replacement (TAVR) has initially been developed for the treatment of severe aortic stenosis in patients deemed at high-risk for conventional surgical approach (1). Technical and procedural improvements in the last years have now expanded its use to lower-risk patients (2).

Even if the incidence of major complications has decreased over the years, conduction disturbances such as high degree atrioventricular block (AVB) or new-onset left bundle branch block (LBBB) remain relatively common (3). Despite an incidence of about one-fourth, the management of new-onset LBBB remains a matter of debate. Its association with increased all-cause and cardiovascular mortality, progression to high degree AVB and need for PM implantation has been shown (3, 4), but the lack of consensus and guidelines has led to substantial heterogeneities in practice. One of the unresolved issues pertains to the exact role of electrophysiological study (EPS) in patients with conduction disturbances post-TAVR. Despite some conflicting results, studies have suggested that performing an EPS after TAVR may be a useful strategy to identify patients who truly need PM implantation in case of new-onset LBBB (5–8). Based on these evidences, a recent scientific expert panel document (3) stated that *an EPS may be a reasonable option in patients with new-onset LBBB, or ECG changes with pre-existing conduction disturbances, when either the QRS or the PR interval exceeds 150 and 240 ms, respectively.*

In order to better define the role of EPS and to optimize its yield, the aim of the present study is to investigate the correlation between post-procedural PR and QRS changes and abnormal HV interval findings during EPS in patients with new-onset LBBB after TAVR. The study is based on the simple assumption that, in case of QRS prolongation, the HV interval should remain normal as long as one fascicle conducts normally, while an abnormal HV interval should imply a PR interval modification (perceptible or not). Accordingly, the hypothesis is that in new-onset LBBB, the analysis of the PR interval may identify more specifically patients with prolonged HV conduction compared to the analysis of the QRS complex.

## Materials and methods

### Design and study population

This is an observational study conducted in two Swiss hospitals including patients with new-onset LBBB post-TAVR. All consecutive patients who underwent an EPS after TAVR between April 2015 and December 2020 were included. Exclusion criteria for analysis were atrial fibrillation/flutter during EPS, previously implanted PM and any type of persistent AVB post-TAVR requiring pacemaker implantation.

Intraventricular conduction disturbances were defined according to the criteria approved by the American Heart Association (9). TAVR procedure were performed using the self-expandable Evolut R and Evolut R Pro (Medtronic, Minneapolis, MN), and the balloon-expandable Sapien 3, (Edwards Life Science, Irvine, CA).

Written informed consent was obtained from all patients and the study was approved by the local ethics committee (Cantonal Ethics Committee Vaud, CER-VD).

### Electrophysiological study and electrocardiogram analysis

EPS was systematically performed in patients with persistent new-onset LBBB post-TAVR as part of our standard tailored management strategy.

The EPS assessment was performed either during the TAVR procedure or within the following days after the procedure in patients with persisting conduction abnormalities. For patients who underwent an HV interval assessment both during and after the TAVR procedure, the second EPS was considered for the analysis.

One or two quadripolar diagnostic catheters were percutaneously inserted through the femoral vein (electrode spacing 5-5-5 mm, 4 mm electrode tip size, Supreme SJN, St. Jude Medical^®^, St Paul, MN). Surface ECG and bipolar intracardiac electrograms were monitored continuously and stored on a computer-based digital amplifier/recorder system (Axiom Sensis XP^®^, Siemens, Berlin, Germany and EPTracer^®^, Cardiotek, Maastricht, Netherlands). Bipolar electrograms were sampled at 2 kHz and band-pass filtered from 30 to 400 Hz. The 12-lead ECG recorded during the EPS was analysed at 100 mm/s sweep speed, with a standard gain of 1 mV/cm and a filter setting of 0.05 Hz (high pass)-100 Hz (low pass). The quadripolar diagnostic catheter was positioned at the most proximal His potential to measure the AH and HV intervals. The mean value of 3 measurements was used. Care was taken to rule out abnormal His potentials suggestive of intra-His conduction delay.

To reproduce real life conditions, the baseline ECG used for analysis the day before the TAVR procedure was recorded on a standard electrocardiograph (Schiller AG, Baar, Switzerland). The ECG was analysed at 50 mm/s sweep speed. Two investigators blinded to the EPS results independently analysed the ECG. In case of disagreement, a consensus was obtained with a third senior investigator.

The analysis was performed using two different cut-offs to define a pathologic HV interval: > 55 and > 60 ms.

### Statistical analysis

Categorical variables are expressed as frequencies (%) and continuous variables as mean ± standard deviation (SD) or median [interquartile range (IQR)] where indicated. Continuous variables were compared by two-tailed paired *t*-test or Mann–Whitney *U*-test in case of abnormal distribution. Categorical variables were tested using Chi-squared tests.

A logistic regression model was used to assess the interdependence of HV interval impairment and ECG prognostic factors. Univariate analyses were performed to reveal unadjusted significant associations between ECG variables and prolonged HV. These variables were entered in the multivariate model to assess adjusted associations between outcomes and covariates.

Receiver-operating characteristic (ROC) curves were generated using the presence of a prolonged HV interval as endpoint: area under the curve (AUC) comparisons were made and the optimal cutoff value was chosen using the Youden Index.

Statistical analysis was carried out using SPSS 24.0 software (SPSS Inc., Chicago, IL), or Matlab (Mathworks, Natick, MA, United States) and 2-sided *p*-values < 0.05 were considered statistically significant.

## Results

### Patients’ characteristics

A total of 78 consecutive patients who developed new-onset LBBB post-TAVR between April 2015 and December 2020 were considered for inclusion. Of those, 17 (21.8%) were excluded due to atrial fibrillation or atrial flutter. Thus, the analysis was performed on a final set of 61 patients. The median age of the population was 81 [76–86] years and 25 patients (41%) were males. Balloon- and self-expandable valves were used in 43 (70.5%) and 18 (29.5%) patients, respectively. The EPS was performed during the TAVR procedure in 26 patients, and 2–10 days following the procedure in 35 patients (median time 3 [2–6] days). Patients’ characteristics are summarized in Table 1.

**TABLE 1:** General characteristics and medical history.

	All patients (*n* = 61)
Age median [IQR]	81 [76–86]
Sex: male	25 (41%)
BMI; Mean ± SD	26 ± 4
Hypertension; *n* (%)	44 (72.1%)
Dyslipidaemia; *n* (%)	32 (52.5%)
Diabetes; *n* (%)	16 (26.2%)
History of atrial fibrillation; *n* (%)	13 (21.3%)
Previous stroke; *n* (%)	8 (13.1%)
CAD; *n* (%)	20 (32.8%)
Chronic renal failure; *n* (%)	15 (24.6%)
Smoking history; *n* (%)	10 (16.4%)
LVEF; Mean ± SD	62 ± 9
Type of valve Balloon-Expandable; *n* (%) Self-Expandable; *n* (%)	43 (70.5%) 18 (29.5%)

### Surface electrocardiogram and HV-interval assessment

The PR and QRS interval pre-TAVR were 185 ± 35 and 96 ± 11 ms, respectively. The PR interval increased to 206 ± 41 ms and the QRS widened to 146 ± 13 ms post-TAVR. A PR interval > 200 ms was observed in 35 patients (57.4%). The ΔPR, defined as the difference between PR interval pre- and post-TAVR was ≥ 20 ms in 27 (44.3%) patients. QRS duration was > 150ms in 26 (42.6%) patients. Regarding the QRS axis, a deviation to the left was observed post-TAVR. A total of 23 patients (37.7%) presented a new left-axis deviation post-TAVR – moderate (between −30° and −45°) in 20 (32.8%) patients, and extreme (beyond −45°) in 3 (4.9%) patients. The pre- and post-TAVR ECG findings are summarized in Table 2.

**TABLE 2:** ECG findings before and after TAVR according to the HV interval assessment.

		Cut-Off HV interval: 55 ms			Cut-Off HV interval: 60 ms		
			
	All patients (*n* = 61)	HV ≤ 55 (*n* = 33)	HV > 55 (*n* = 28)	*P-value*	HV ≤ 60 (*n* = 44)	HV > 60 (*n* = 17)	*P-value*
**PR interval** PR Pre; Mean (IQR) PR Post; Mean ± SD PR interval > 200 ms; *n* (%) ΔPR; Mean ± SD	185 ± 35 206 ± 41 35 21 ± 29	178 ± 35 188 ± 38 10 10 ± 30	194 ± 33 228 ± 34 25 34 ± 23	0.059 <0.001 <0.001 0.001	182 ± 34 197 ± 40 19 16 ± 31	195 ± 36 229 ± 34 16 34 ± 21	0.187 0.006 <0.001 0.024
**QRS interval** QRS Pre; Mean ± SD QRS Post; Mean ± SD QRS interval > 150 ms; *n* (%) ΔQRS; Median (IQR)	96 ± 11 146 ± 13 26 53 [43–59]	95 ± 11 145 ± 14 14 52 [42–60]	97 ± 11 148 ± 13 12 53 [44–57]	0.413 0.355 0.973 0.546	95 ± 12 145 ± 14 19 52 [42–59]	98 ± 10 149 ± 10 7 53 [42–59]	0.333 0.235 0.887 0.778
**QRS Axis** QRS Axis Pre; Median (IQR) QRS Axis Post; Median (IQR) New Left Axis Deviation post-TAVR; *n* (%) ΔAxis; Mean ± SD	0° [−15° to 30°] −15° [−45° to 15°] 23 −20° ± 41	15° [−15° to 30°] −15 [−45° to 38°] 10 −14° ± 41°	−8° [−15° to 16°] -45 [−45° to -4°] 13 −27° ± 42°	0.144 0.037 0.195 0.222	8° [−15° to 30°] −15° [−45° to 26°] 14 −15° ± 42°	0° [−23° to 23°] −45° [−45° to −8°] 9 −34° ± 38°	0.435 0.052 0.127 0.097

The median HV interval duration post-TAVR in new-onset LBBB was 54 [50–65] ms. An abnormal HV interval exceeding the 55 ms or 60 ms cut-off values was found in 28 (45.9%) and 17 (27.9%) patients, respectively. An HV interval > 70 ms was found in 9 (14.8%) patients.

### HV interval assessment according to the *PR interval*

The post-TAVR PR and ΔPR interval durations were significantly longer in patients with an HV interval > 55 ms post-TAVR (228 ± 34 vs. 188 ± 38 ms, *p* < 0.001 for the PR interval; and 34 ± 23 vs. 10 ± 30 ms, *p* = 0.001 for the ΔPR interval). Similar findings were observed when considering an HV interval cut-off of 60 ms (229 ± 34 vs. 197 ± 40 ms, *p* = 0.006 for the PR interval, 34 ± 21 vs. 16 ± 31 ms, *p* = 0.024 for the ΔPR interval). The pre-implantation baseline PR interval did not show a statistically significant difference between patients with normal and prolonged HV interval independently from the considered cut-off. The HV interval assessment according to the PR interval are summarized in Table 2.

### HV interval assessment according to *QRS duration and axis*

The QRS and ΔQRS duration post-TAVR did not differ significantly between patients with normal and abnormal HV interval using both a 55 or 60 ms cut-off values. Regarding the QRS axis, ΔAxis and the occurrence of a new left axis deviation did not differ significantly between both groups for both HV interval cut-off values. The HV interval assessment according to the post-TAVR QRS duration and axis are summarized in Table 2.

### Proposed electrocardiogram cut-off values to predict abnormal electrophysiological findings

**FIGURE 1:**
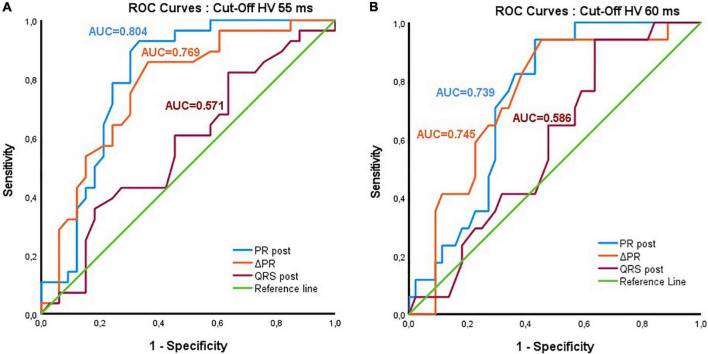
ROC curves for PR, ΔPR and QRS intervals to discriminate patients with abnormal HV after transcatheter aortic valve replacement: **(A)** HV interval cut-off 55 ms; **(B)** HV interval cut-off 60 ms.

The ROC curve analysis to discriminate patients with an HV interval exceeding 55 ms yielded an optimal cut-off for the PR interval post-TAVR of 199.5 ms (Sensitivity = 92.9%, Specificity = 66.7%, Youden Index = 0.595; AUC = 0.804, *p* < 0.001). The optimal ΔPR interval for the same cut-off was > 13 ms (Sensitivity = 85.7%, Specificity = 63.6%, Youden Index = 0.494; AUC = 0.769, *p* < 0.001; Figure 1A).

For the 60 ms HV cut-off value, the analysis yielded an optimal cut-off of > 202 ms for the PR interval (Sensitivity = 94.1%, Specificity = 56.8%, Youden Index = 0.509; AUC = 0.739, *p* = 0.004), and > 13 ms for ΔPR (Sensitivity = 94.1%, Specificity = 54.5%, Youden Index = 0.487; AUC = 0.745, *p* = 0.003) (Figure 1B).

In order to provide ECG cut-off values that can be used readily in clinical practice, considering the difficulty to measure lower than 20 ms intervals on standard ECG recordings, a PR interval > 200 ms and a ΔPR interval ≥ 20 ms were used for further analysis.

### Prediction of abnormal HV interval based on the electrocardiogram findings

On univariate analysis, the presence of PR interval > 200 ms post-TAVR was predictive of a prolonged HV interval, both for the 55 and 60 ms cut-offs (OR: 19.2, 95% CI: 4.7–78.4, *p* < 0.001 and OR: 21.1, 95% CI: 2.6–173.0, *p* = 0.005, respectively). Regarding the PR interval change post-TAVR, a ΔPR interval ≥ 20 ms predicted both an HV interval > 55 and 60 ms (OR: 4.8, 95% CI: 1.6–14.3, *p* = 0.005, and OR: 4.6, 95% CI: 1.4–15.6, *p* = 0.013, respectively).

Importantly, neither a QRS interval > 150 ms nor a new left axis deviation post-TAVR predicted abnormal EP results using both cut-offs.

On multivariate analysis, a PR interval > 200 ms was the only factor independently associated with a prolonged HV interval for both a 55 and 60 ms cut-offs (OR: 18.0, 95% CI 3.9–83.4, *p* < 0.001 and OR: 16.7, 95% CI: 1.9–146.2, *p* = 0.011, respectively). Univariate and multivariate analyses are presented in Table 3.

**TABLE 3:** Prediction of abnormal HV post-implantation based on the electrocardiogram.

Cut-Off HV interval: 55 ms							

				Univariate analysis		Multivariate analysis	
					
	Total	HV ≤ 55	HV > 55	OR (95% CI)	*P-value*	OR (95% CI)	*P-value*
Patients	61	33	28				
PR interval > 200 ms	35	10	25	19.2 (4.7–78.4)	<0.001	18.0 (3.9–83.4)	<0.001
ΔPR ≥ 20 ms	27	9	18	4.8 (1.6–14.3)	0.005	3.6 (0.9–13.5)	0.059
QRS interval > 150 ms	26	14	12	1.0 (0.4–2.8)	0.973	1.8 (0.4–7.3)	0.413
New left axis deviation	23	10	13	2.0 (0.7–5.7)	0.198	1.6 (0.4–6.6)	0.482
**Cut-Off HV interval: 60 ms**							
				**Univariate analysis**		**Multivariate analysis**	
					
	**Total**	**HV ≤ 60**	**HV > 60**	**OR (95% CI)**	***P-value***	**OR (95% CI)**	***P-value***

Patients	61	44	17				
PR interval > 200 ms	35	19	16	21.1 (2.6–173.0)	0.005	16.7 (1.9–146.2)	0.011
ΔPR ≥ 20 ms	27	15	12	4.6 (1.4–15.6)	0.013	3.1 (0.8–12.3)	0.108
QRS interval > 150 ms	26	19	7	0.9 (0.3–2.9)	0.887	1.4 (0.3–5.6)	0.661
New left axis deviation	23	14	9	2.4 (0.8–7.6)	0.132	1.9 (0.5–7.4)	0.349

### Predictive value of PR interval assessment to predict abnormal HV interval

A PR interval exceeding 200 ms provided an 89% sensitivity and an 88% negative predictive value (NPV) (specificity = 70%, positive predictive value (PPV) = 71%) to identify patients with an HV interval exceeding 55 ms. When using a 60 ms cut-off value, the sensitivity and NPV increased to 94 and 96%, respectively (specificity = 57%, PPV = 46%).

A ΔPR ≥ 20 ms provided a somewhat lower sensitivity (64%) and NPV (71%) for the HV cut-off of 55 ms (specificity = 73%, PPV = 67%). For the 60 ms cut-off value, sensitivity was 71%, while the NPV was 85% (specificity = 66%, PPV = 44%).

### Combined use of PR and ΔPR interval to predict a prolonged HV interval

**FIGURE 2:**
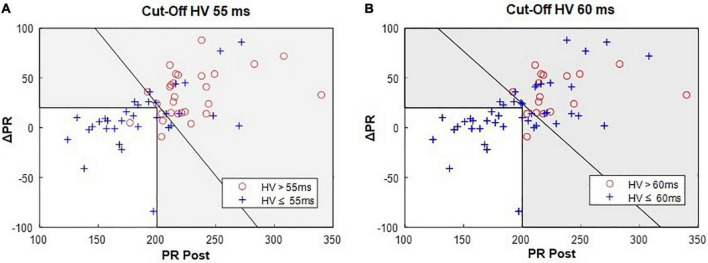
Bivariate analysis of the PR and ΔPR intervals: 96% (27/28) of patients with an HV > 55 ms **(A)** have PR > 200 ms OR ΔPR ≥ 20 ms; **(B)** 100% (17/17) of patients with an HV > 60 ms have PR > 200 ms OR ΔPR ≥ 20 ms. In each graph, the oblique line indicates the optimal separation between normal and abnormal HV intervals. Gray area represents the acceptance zone for the parallel testing, i.e., PR post > 200 ms OR ΔPR ≥ 20 ms.

The combined use of *either* an abnormal PR *or* ΔPR interval, allowed a notable increase in sensitivity to discriminate patients with abnormal HV interval. The finding of a PR interval > 200 ms *or* a ΔPR interval ≥ 20 ms, yielded a 96% sensitivity and 95% NPV (specificity = 55%, PPV = 64%) to identify patients with an HV interval exceeding 55 ms (Figure 2A). The only missed case was a patient with a borderline HV interval (58 ms). Accordingly, using this combined assessment with an HV interval cut-off of 60 ms identified all patients with abnormal EP results (sensitivity and NPV of 100%, specificity = 43%, PPV = 40%; Figure 2B). A selective strategy which would consist in performing EPS only in case of an abnormal PR *or* ΔPR interval, would avoid 19 (31%) exams in our study population with a PPV of 64%.

On the other hand, considering the combined use of both an abnormal PR *and* ΔPR interval increased the specificity at the cost of a lower sensitivity. Thus, the finding of both a PR interval > 200 ms *and* a ΔPR interval ≥ 20 ms yielded a specificity of 88% and a PPV of 80% (Sensitivity = 57%, NPV = 71%) for the 55 ms HV cut-off value. Similar results were found for the HV cut-off of 60 ms.

The performance of the combined use of PR and ΔPR interval to predict a prolonged HV interval is summarized in Table 3.

## Discussion

### Main findings

The major finding of the present study is the identification of ECG parameters which allow selecting patients with new-onset LBBB after TAVR who may benefit most from performing an EPS in order to rationalize its use. In patients with a post-TAVR PR interval ≤ 200 ms *and* a ΔPR < 20 ms, an EPS will have an extremely low yield and may therefore be avoided. Importantly, these findings hold true independently of the QRS changes in duration or axis.

In this study population, the use of the proposed PR interval assessment to selectively perform an EPS would avoid about one third of exams in patients with new-onset LBBB without missing any patients with significantly prolonged HV interval (i.e., ≥ 60 ms). The PPV of such strategy would be 64%.

### Role of the electrophysiological study in new-onset left bundle branch block

The lack of guidelines in the management of patients with new-onset LBBB after TAVR has led to substantial heterogeneities in practices. Indications for PM implantation are currently tailored individually based on either the 12-lead ECG alone (e.g., based on PR interval and/or QRS duration) (10, 11), or the results of EP testing (6–8, 12). More recently, Knecht et al. showed that a management strategy based on a simple HV interval measurement performed with the temporary pacemaker wire could safely identify patients with LBBB who will not develop high degree AVB with a NPV of 90% (6). A recent scientific expert panel state that an EPS was a reasonable option in patients with new-onset LBBB when either the QRS or the PR interval exceeds 150 and 240 ms, respectively (3). The present study adds on accumulated evidences showing that a management strategy based on EP testing should rely on the absolute PR value and its changes, but not on the QRS duration, in order to select the best candidates for EP testing.

In new-onset LBBB, a tailored strategy based on the PR interval assessment may help rationalize resource utilization and hospitalization length without compromising safety.

### HV interval cut-off

In the present study, we analysed two different cut-off values to define a pathologic HV interval, namely > 55 and > 60 ms. These two cut-off values are the most stringent that have been used by some groups to justify prophylactic PM implantation (6, 7, 13). Nevertheless, in most previous studies as well as in the above-mentioned expert panel and the latest ECG Guidelines on cardiac pacing, higher cut-offs have generally been used to justify PM implantation, ranging from 70 to 100 ms (3, 5, 8, 12, 14–16). Accordingly, a strategy relying on a selective use of EPS that is able to identify the vast majority of patients with an HV interval above these more stringent cut-offs should likely be safe. The recent data by Knecht et al. (6) support this hypothesis; they showed that an HV interval ≤ 55 ms assessed within 24h of the TAVR procedure identified patients with LBBB who did not develop high-grade AVB with a NPV of 90%. Our proposed strategy combining the PR and ΔPR interval assessment, identified patients above this 55 ms cut-off with a 95% NPV. The NPV was 100% for an HV cut-off of 60 ms. This cut-off may be more relevant for clinical decision making, at least in terms of prophylactic PM implantation, since it is more in the range of the values generally used by most groups to justify prophylactic PM implantation. Rivard et al. (7) showed that in patients with new-onset LBBB, a postprocedural HV interval ≥ 65 ms predicted AVB with 83% sensitivity and 82% specificity. Similarly, a recent review of the literature on EPS after TAVR suggested that EPS-guided PM implantation should be based on HV interval values in the range of 65–75 ms or more (17). Finally, from an electrophysiological standpoint, it is worth noting that in the setting of LBBB, some authors (18) believe that 60 ms is a more appropriate upper limit of normal HV interval. Indeed, considering that the left side of the septum is normally activated earlier by the left bundle branch, differences of 5–15 ms in the HV interval are sometimes observed with the development of LBBB despite intact right bundle branch conduction (19).

### Analysis of the PR interval to predict the risk of atrioventricular block and abnormal electrophysiological study findings

The relevance of the PR interval assessment to stratify the risk of advanced AVB and abnormal EPS findings has been reported by other groups (10–12, 14, 20, 21). Akin et al. showed that new-onset LBBB with PR interval > 200 ms post-TAVR was predictive of high-grade AVB, and 18 of the 22 patients suffering from first-degree AVB demonstrated prolonged HV interval. Toggweiler et al. (11) and Jorgensen et al. (10) both evaluated predictors of delayed high-degree AVB occurring within 30 days of the TAVR procedure in a total population of about 1500 patients. They both demonstrated a similar association with first-degree AVB post-TAVR and the risk of subsequent high-degree AVB. In the study by Toggweiler et al., the proportion of high-grade AVB was 6.8 and 15.7% in patients with LBBB with and without first degree AVB, respectively (*p* < 0.001).

Regarding the relevance of assessing the pre- and postprocedural PR interval changes, Tovia et al. found that, out of 24 patients with LBBB, none of the patients without post procedural PR prolongation, using a ΔPR interval cut-off > 20 ms as proposed in our study, had significant infranodal disease (12). Mangieri et al. showed that among 611 patients after TAVR, the two independent predictors of late PM implantation (≥48 h) were baseline RBBB, and the amount of PR prolongation post-TAVR (OR for each 10 ms increments: 1.31; 95% CI: 1.18–1.45; *p* < 0.001) (21). Of note, the reported mean ΔPR interval in patients requiring PM implantation was consistently of about 40 ms among studies that reported this variable (20, 21).

Considering the aim of our proposed strategy to limit the number of EPS without missing patients with abnormal HV interval, the above-mentioned evidences tend to support an EPS selection process incorporating both the PR (10, 11, 14), and the ΔPR interval (12, 20, 21).

### QRS duration to predict the risk of atrioventricular block and abnormal electrophysiological study findings

Among patients with new-onset LBBB, we did not find any correlation between the QRS interval and abnormal HV interval at EPS. To our knowledge, there are no data available addressing the correlation between the QRS interval (beyond 120 ms) and the HV interval in new-onset LBBB after TAVR. Furthermore, only limited data showed that, in new-onset LBBB, a longer QRS duration (i.e., >150–160 ms) may be associated with an increased risk of delayed high-degree AVB compared to a relatively narrower QRS *irrespective of the PR interval*. Urena et al. found that in patients with new-onset LBBB and a QRS interval > 160 ms at discharge, the risk of sudden cardiac death was significantly increased (9.9 vs. 3% in patients with new-onset LBBB and QRS-interval ≤ 160 ms), suggesting a higher rate of advanced heart block in these patients as an etiology. This assumption was based on the fact that no increased risk of SCD was observed in patients with new-onset LBBB and PM implantation before discharge (4). On the other hand, Jorgensen et al. provided some more direct evidence showing that high-degree AVB with insufficient escape rhythm only occurred with longer QRS duration (≥150 ms) in patients in sinus rhythm with LBBB (7.1%; 95% CI 2.6–14.7%) (10).

## Study limitations

The proposed strategy to select EPS candidate should be validated in a separate and larger patient population. Moreover, the aim of the study was to provide a key to rationalize the use of EPS in patients with new-onset LBBB post-TAVR but it did not evaluate the ability of the EPS to identify patients at risk of AVB. Further studies are needed for this purpose.

The yield of the EPS was considered exclusively on the basis of the basal HV interval assessment but other maneuvers may further stratify the risk of AVB. The use of incremental atrial pacing or pharmacological challenge (such as ajmaline or procainamide) to stress the His-Purkinje system would have possibly revealed additional patients at risk of AVB despite normal basal HV interval. The proportion of such patients is, however, expected to be limited. It was indeed observed in one out of the 35 patients who underwent a comprehensive EP evaluation.

In our study, the assessment of the HV interval was performed early post-TAVR in a significant subset of patients, while it has been suggested that EPS is best performed 3 days or more after TAVR and after conduction abnormalities have stabilized (16, 22). Nevertheless, since the aim was to correlate the surface ECG to the HV assessment at a given moment, we think that this limitation does not significantly affect the applicability of our findings.

Finally, since our strategy is based on the PR interval assessment, it cannot be implemented in patients with AF which represent about one fifth of patients, both in our study and in previous studies (6, 10, 11).

## Conclusion

The PR interval assessment in patients with new-onset LBBB after TAVR may be a useful simple tool to select patients who may benefit most from an EPS and rationalize its use. Namely, for patients with a post-TAVR PR interval ≤ 200 ms, and a ΔPR < 20 ms, an EPS will have an extremely low yield independently of QRS changes.

## Data availability statement

The raw data supporting the conclusions of this article will be made available by the authors upon reasonable request.

## Ethics statement

The studies involving human participants were reviewed and approved by Cantonal Ethics Committee Vaud, CER-VD. The patients/participants provided their written informed consent to participate in this study.

## Author contributions

MP, DM, PP, OM, and SF were involved in both the conception of the study and analysis of the data. SC, J-JG, MD, VR, CR, AL, FA, PG, CH, GD, ML, CH-S, GG, EP, and EE contributed to the analysis and interpretation of data. All authors contributed substantially to the realization of this study and participated in drafting and revising the manuscript and gave their approval to the submitted version.

## Abbreviations

AUC: area under the curve
AVB: atrioventricular block
ECG: electrocardiogram
EPS: electrophysiological study
IQR: interquartile range
LBBB: left bundle branch block
NPV: negative predictive value
OR: odd ratio
PPV: positive predictive value
PM: pacemaker
RBBB: right bundle branch block
ROC: receiver-operating characteristic
SD: standard deviation
TAVR: trans-catheter aortic valve replacement.
