# Corrigendum: Yield of the electrophysiological study in patients with new-onset left bundle branch block after transcathether aortic valve replacement: The PR interval matters

**DOI:** 10.3389/fcvm.2022.1065221

**Published:** 2022-10-18

**Authors:** Mattia Pagnoni, David Meier, Adrian Luca, Stephane Fournier, Farhang Aminfar, Pascale Gentil, Christelle Haddad, Giulia Domenichini, Mathieu Le Bloa, Claudia Herrera-Siklody, Stephane Cook, Jean-Jacques Goy, Christan Roguelov, Grégoire Girod, Vladimir Rubimbura, Marion Dupré, Eric Eeckhout, Etienne Pruvot, Olivier Muller, Patrizio Pascale

**Affiliations:** ^1^Department of Cardiology, Lausanne University Hospital, Lausanne, Switzerland; ^2^Division of Cardiology, Department of Advanced Biomedical Sciences, University of Naples Federico II, Naples, Italy; ^3^Arrhythmias Unit, Louis Pradel Cardiovascular Hospital, Hospices Civils de Lyon, Lyon, France; ^4^Department of Cardiology, Clinique Cecil Hirslanden Group, Lausanne, Switzerland; ^5^Department of Cardiology, University Hospital Fribourg, Fribourg, Switzerland

**Keywords:** electrophysiological study (EPS), trans-catheter aortic valve replacement (TAVR), atrioventricular block (AV block), HV interval, PR interval

In the published article, an author name was incorrectly written as “Mathieu Lebloa”. The correct spelling is “Mathieu Le Bloa”.

The authors apologize for this error and state that this does not change the scientific conclusions of the article in any way. The original article has been updated.

## Publisher's note

All claims expressed in this article are solely those of the authors and do not necessarily represent those of their affiliated organizations, or those of the publisher, the editors and the reviewers. Any product that may be evaluated in this article, or claim that may be made by its manufacturer, is not guaranteed or endorsed by the publisher.

